# Supplementary material for: TLR4 is a regulator of trained immunity in a murine model of Duchenne muscular dystrophy
Source: Nat Commun. 2022 Feb 15;13:879. doi: 10.1038/s41467-022-28531-1 (PMC8847425; doi:10.1038/s41467-022-28531-1)
Supplement: Supplementary file 1 — Supplementary Information [file 41467_2022_28531_MOESM1_ESM.pdf]

SUPPLEMENTARY INFORMATION for

TLR4 is a regulator of trained immunity in a murine model of Duchenne muscular dystrophy

Salyan Bhattarai[1,2], Qian Li[1,2], Jun Ding[1,2], Feng Liang[1,2], Ekaterina Gusev[1,2], Orsolya Lapohos[1,2], Gregory J Fonseca[1,2], Eva Kaufmann[1,2], Maziar Divangahi[1,2,3], Basil J Petrof[1,2]

[1] Meakins-Christie Laboratories, Translational Research in Respiratory Diseases Program, Research Institute of the McGill University Health Centre, 1001 Decarie Boulevard, EM3-2219, Montreal, Quebec, Canada H4A 3J1

[2] Department of Medicine, McGill University Health Centre, 1001 Decarie Boulevard, D5, Montreal, Quebec, Canada H4A 3J1

[3] Department of Microbiology and Immunology, McGill University, Duff Medical Building, 3775 University Street, Montreal, Quebec, Canada H3A 2B4

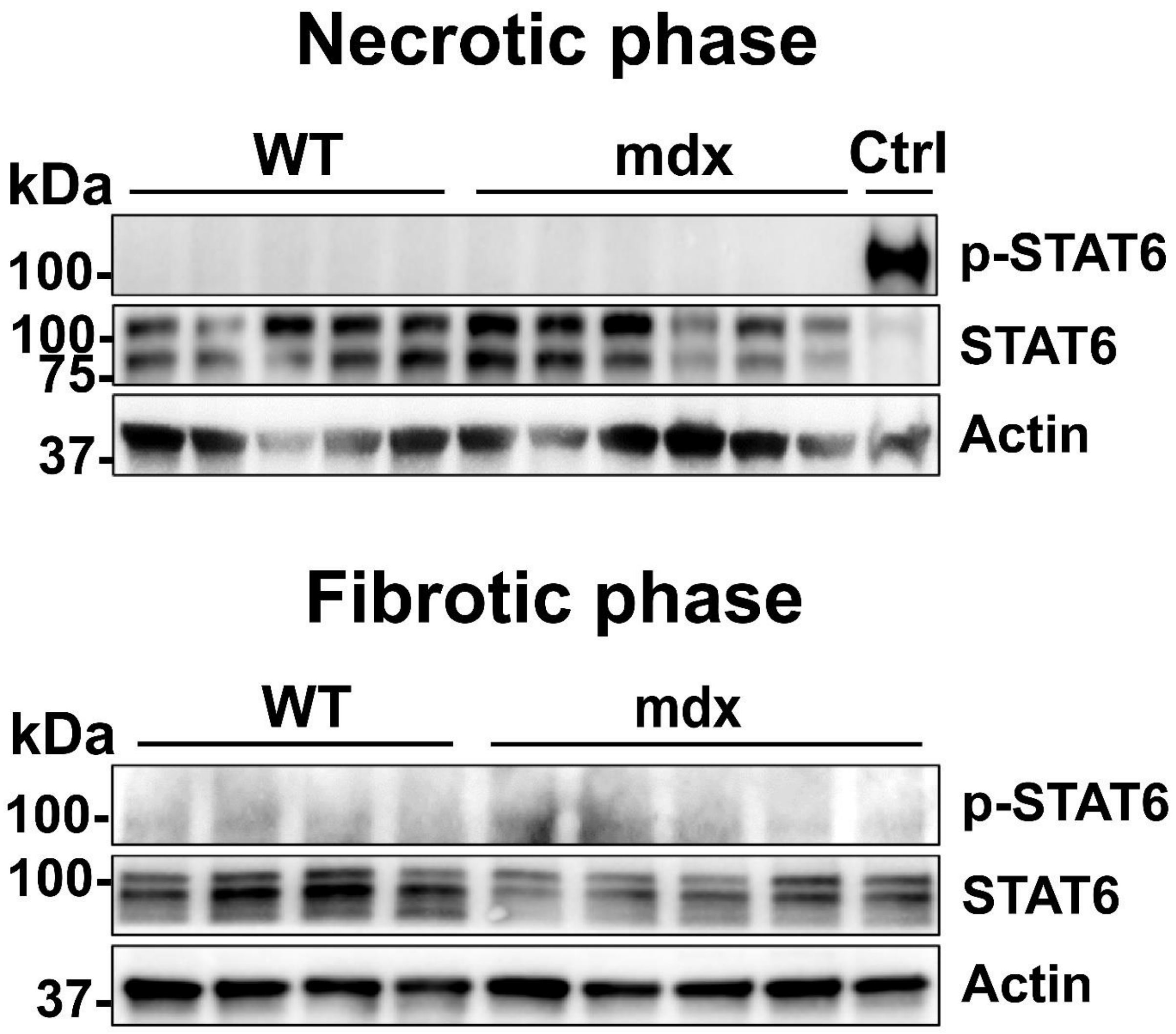

Supplementary Figure 1: Lack of STAT6 phosphorylation in mdx BMDM. Levels of p-STAT6 in total cell lysate of WT and mdx BMDM were evaluated by western blot at necrotic (top panel) and fibrotic (bottom panel) phases of the disease. WT BMDM treated with 20 ng/ml IL4 for 2 hours, served as a positive control (Ctrl, in the top panel). Total STAT6 was detected in all samples, and actin served as an additional loading control. Each lane represents an independent biological sample obtained from a different animal, and all experimental replicates are shown in the figure.

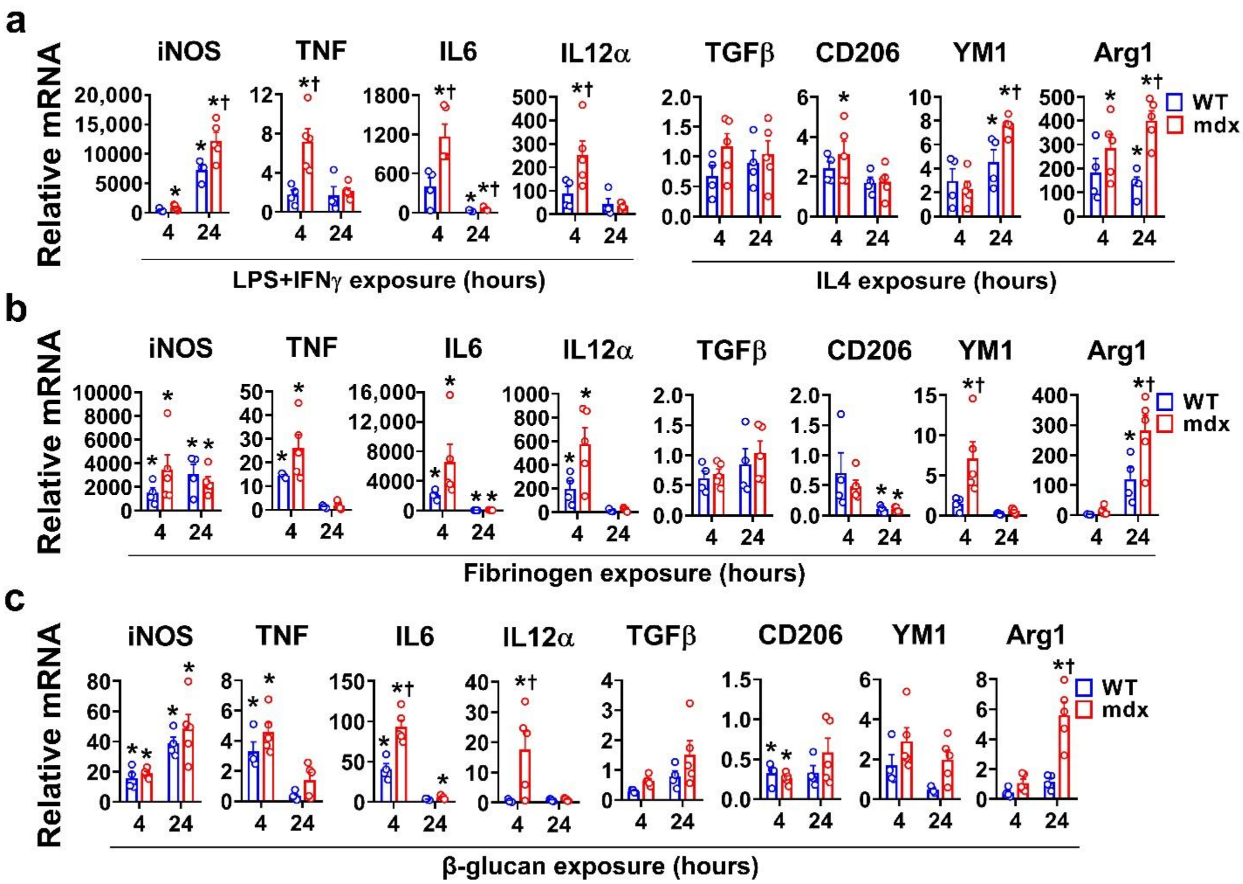

Supplementary Figure 2: mdx BMDM at the fibrotic phase of disease show non-specific amplified responses to heterologous stimuli. BMDM generated from WT and mdx mice at the fibrotic phase of disease were exposed to (**a**) LPS (100 ng/ml) + IFNγ (20 ng/ml) and IL4 (20 ng/ml) (n=4/group for WT, n=5/group for mdx), (**b**) fibrinogen (1 mg/ml) (n=4/group for WT, n=5/group for mdx), or (**c**) β-glucan (100 µg/ml) (n=4/group for WT, n=5/group for mdx except IL6 at 24h n=4). Bar graphs show mRNA transcript levels of prototypical pro-inflammatory ("M1") and anti-inflammatory ("M2") marker genes at 4 and 24 hours after exposure. All values are expressed relative to the mean basal WT (unstimulated) level determined on the same PCR plate. Data represent means ± SEM of biologically independent samples from different mice. *P<0.05 vs unstimulated WT BMDM and †P<0.05 vs stimulated WT BMDM at a given

time point (one-way ANOVA followed by Tukey post-hoc test, two-tailed). See Source Data file for the exact P values.

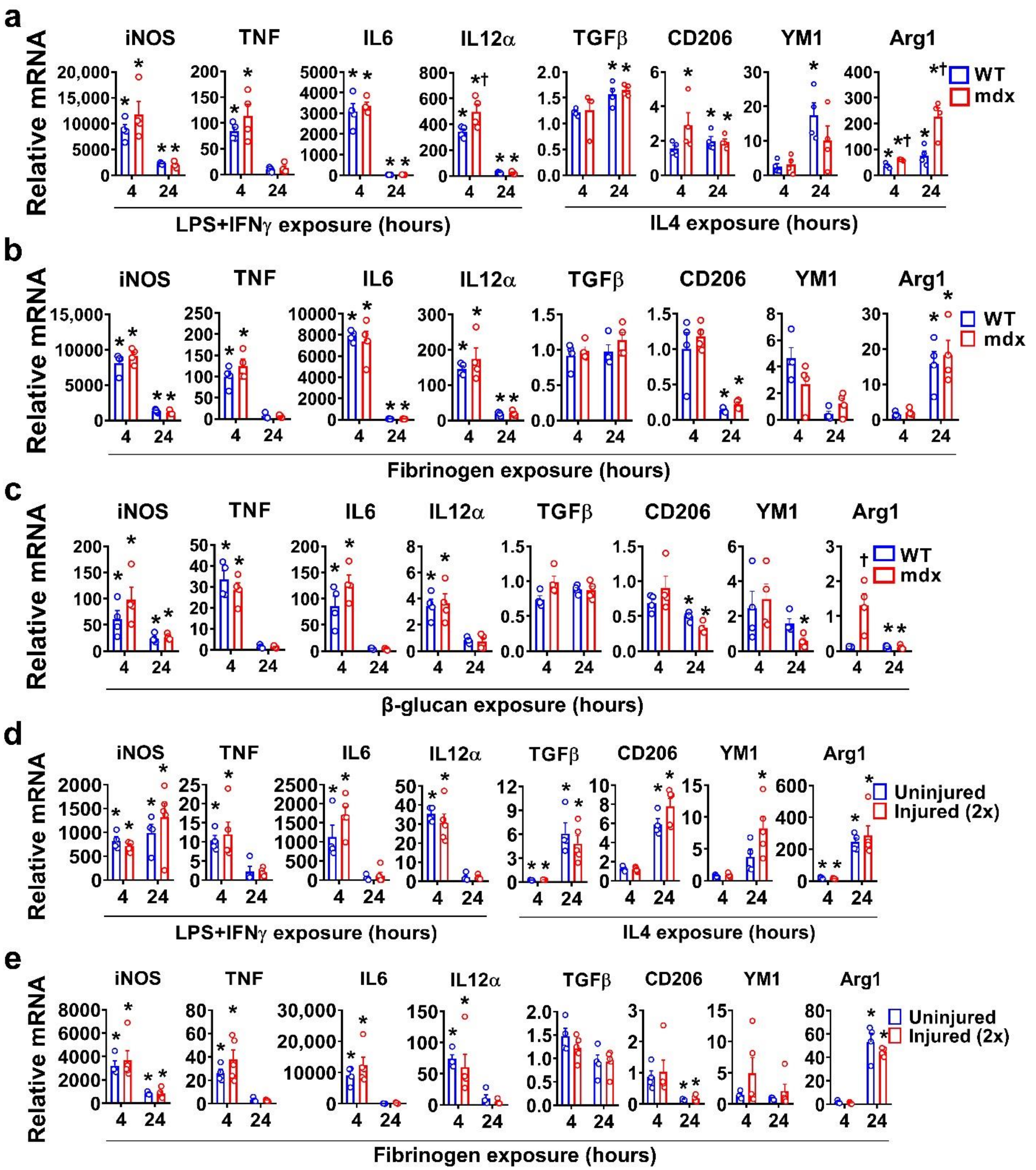

Supplementary Figure 3: BMDM from prenecrotic mdx mice and cardiotoxin-injured mice do not exhibit amplified responses to heterologous stimuli. BMDM from WT and mdx mice at the prenecrotic phase of disease were exposed to (**a**) LPS + IFNγ or IL4 (n=4/group) (**b**) fibrinogen (n=4/group), or (**c**) β-glucan (n=4/group except

TGFβ in mdx 24h group n=3). Bar graphs show mRNA transcript levels of prototypical pro-inflammatory (“M1”) and anti-inflammatory (“M2”) marker genes at 4 and 24 hours after exposure. (**d-e**) BMDM generated from uninjured and cardiotoxin-injured WT were exposed to heterologous stimuli as in (a-c) (n=4/group for Uninjured, n=5/group for Injured). All values are expressed relative to the mean basal WT level determined on the same PCR plate. Data represent means ± SEM of biologically independent samples from different mice. **P*<0.05 vs unstimulated WT/Uninjured BMDM and †*P*<0.05 vs stimulated WT/Uninjured BMDM (one-way ANOVA followed by Tukey post-hoc test, two-tailed). See Source Data file for the exact P values.

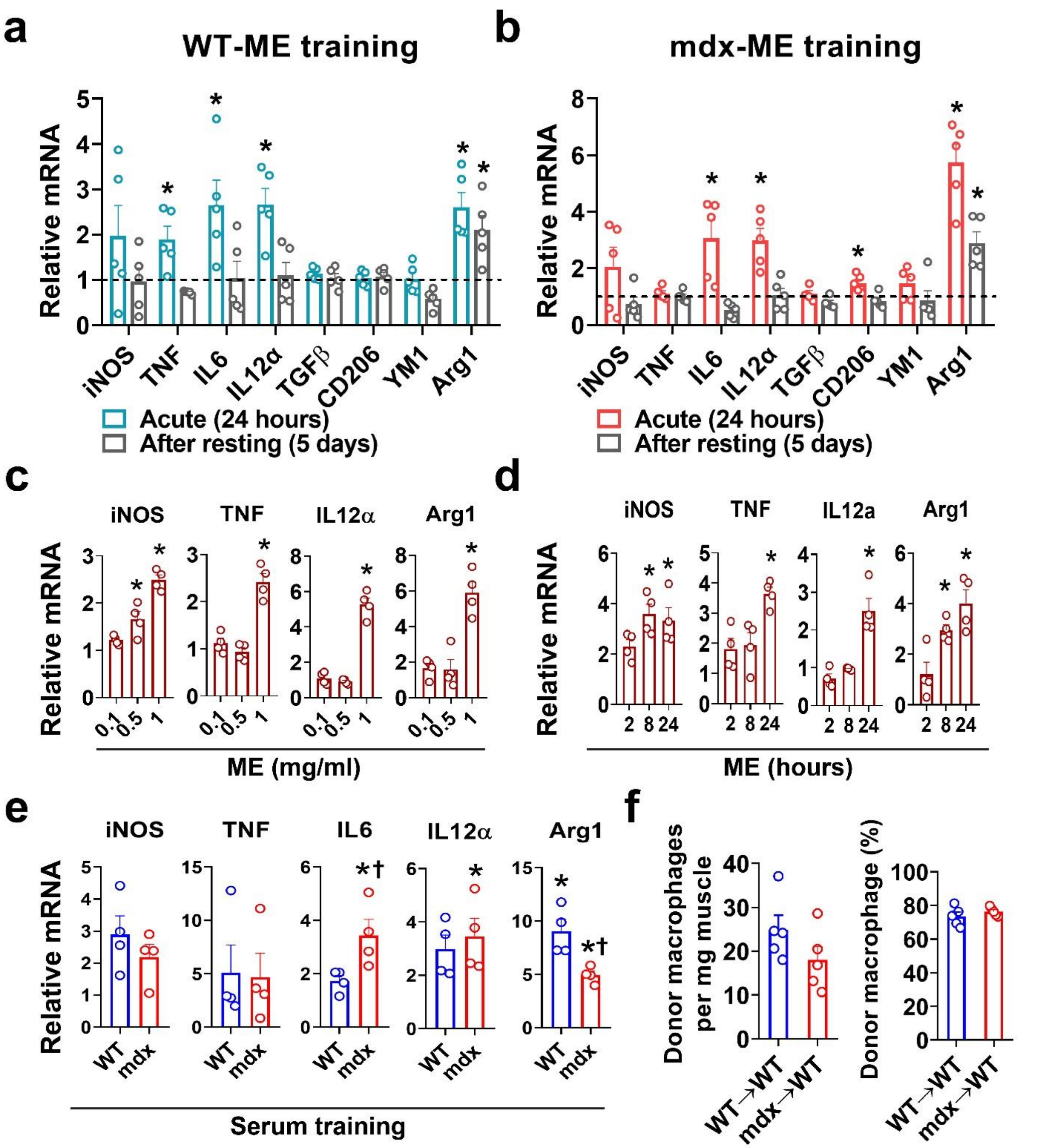

Supplementary Figure 4: M1 and M2 marker gene expression after "training" by muscle extract or serum in vitro. As indicated in Fig. 4a, WT BMDM were exposed for 24 hours to muscle extract (ME) derived from either (**a**) WT (n=5 per group) or (**b**) mdx muscles (n=5/group except for TGFβ at 24h n=4). Gene expression was measured

immediately after ME exposure (24 hours) as well as after removal of the ME stimulus and subsequent “resting” of the cells (5 days). The dashed line indicates the mean basal expression level for PBS-exposed WT BMDM at each time point. (**c-d**) Expression of inflammatory genes 8 hours after fibrinogen exposure in WT BMDM previously “trained” with mdx-ME at (**c**) different concentrations (0.1, 0.5 and 1 mg/ml) for 24 hours (n=4 per group), or (**d**) different exposure durations (2, 8, 24 hours) using the same concentration (1 mg/ml) (n=4 per group). Data are expressed relative to the PBS-trained group identically stimulated with fibrinogen. (**e**) Identically to the ME protocol (Fig. 4a), WT BMDM (n=4 per group) were “trained” with WT or mdx (4-6 weeks old) serum (5%) followed by resting for 5 days and secondary stimulation with fibrinogen for 8 hours. The graph shows the M1 and M2 markers gene expression levels relative to the PBS-trained group stimulated with fibrinogen. (**f**) Donor macrophage (defined as CD45.2+CD11c-CD11b+F4/80+) number (left panel) and percentage (right panel) in the tibialis anterior (TA) muscle of mice (n=5 per group) transplanted with WT or mdx bone marrow as shown in Fig. 4d. There were no differences between groups. All data represent means ± SEM of independent biological or mice (**a**-**b**, **f:** *P<0.05 unpaired t-test, two-tailed; **c**-**e:** *P<0.05 vs. PBS- trained WT BMDM one-way ANOVA followed by Tukey post-hoc test, two-tailed). See Source Data file for the exact P values.

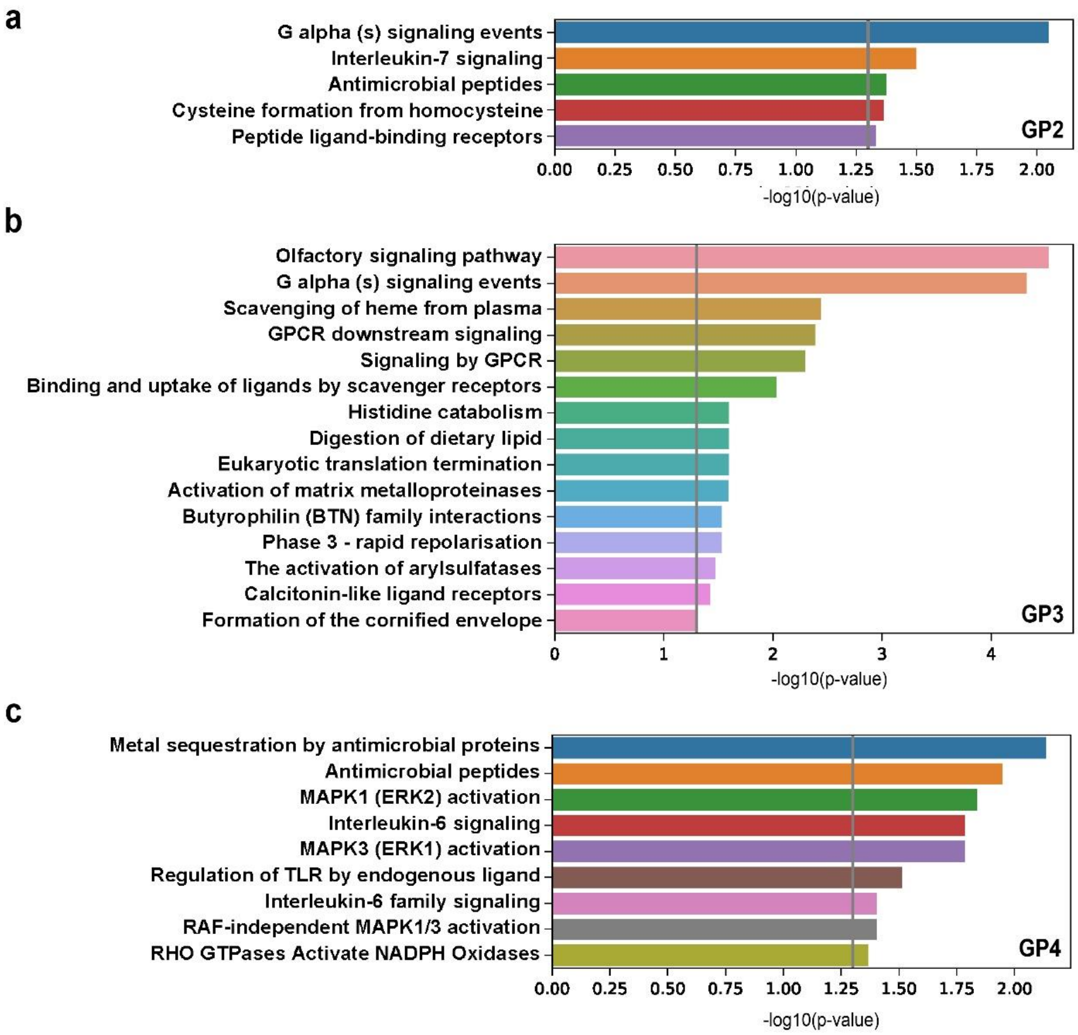

Supplementary Figure 5: Pathway analysis (Reactome database) for the Gene-based Patterns (GP) designated GP2, GP3 and GP4 for H3K27me3. Pathway enrichment analysis is shown for genes showing the (**a**) GP2, (**b**) GP3, and (**c**) GP4 configurations. The vertical line indicates the cut-off P value = 0.05. The extended gene lists are found in the Supplementary Data 2 table.

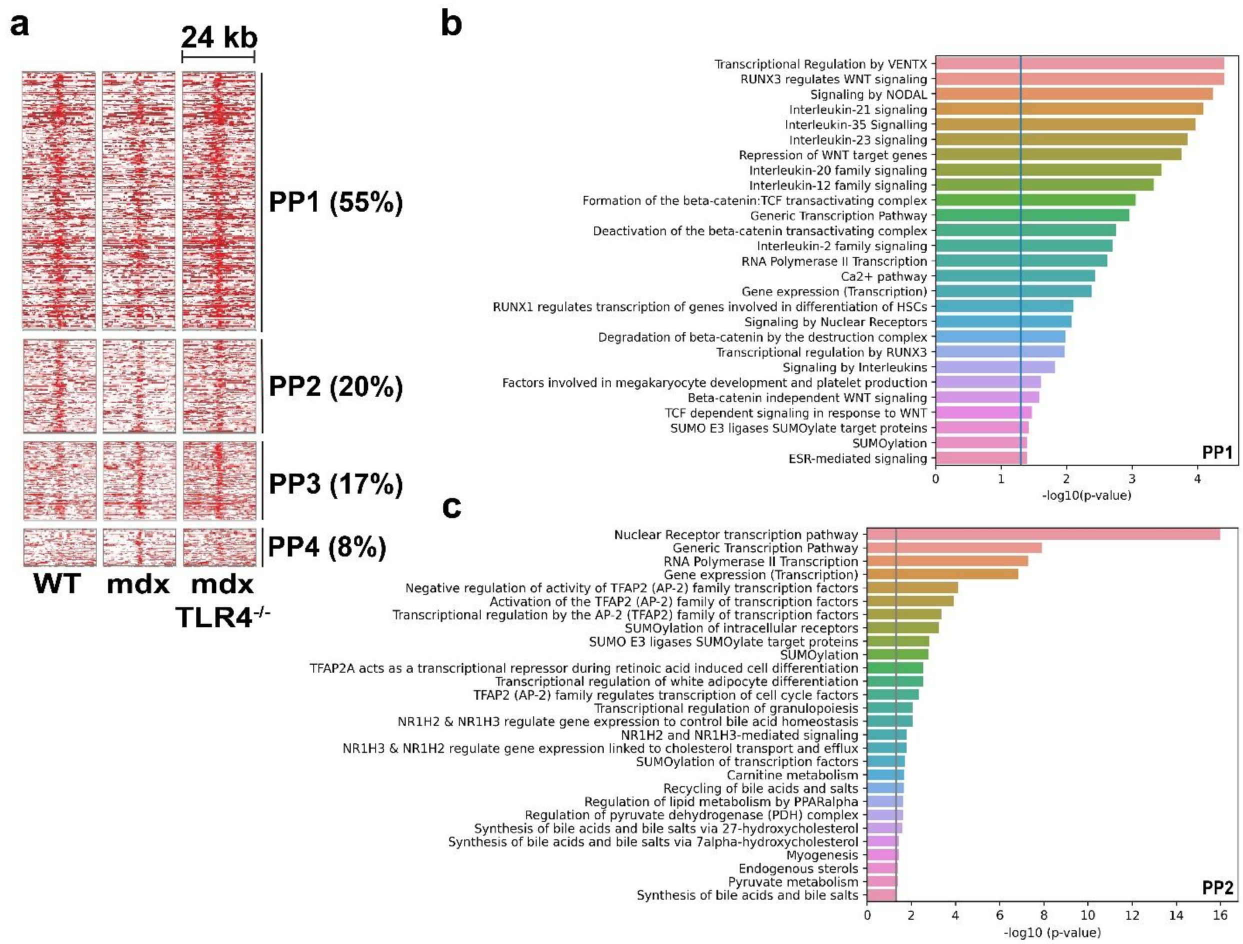

Supplementary Figure 6: Transcription factor enrichment analysis for H3K27me3 in WT, mdx and mdxTLR4$^{-/-}$ BMDM. (**a**) Stacked heatmaps of the Peak-based Pattern (PP) analysis showing the dynamically regulated patterns in mdx versus WT (designated PP1 to PP4); the normalized H3K27me3 read intensity is plotted ±12 kb over the center of peaks. The values in parentheses indicate the percentages for each pattern (I=Increased and U=Unchanged relative to Input) as follows: PP1=IUI, PP2=IUU, PP3=UII and PP4=UIU. (**b**-**c**) Transcription factor enrichment Reactome pathway analysis for (**b**) PP1, and (**c**) PP2 patterns. Vertical lines indicate the cut-off P value = 0.05. The extended lists for these data can be found in Supplementary Data 4 and 5 tables.

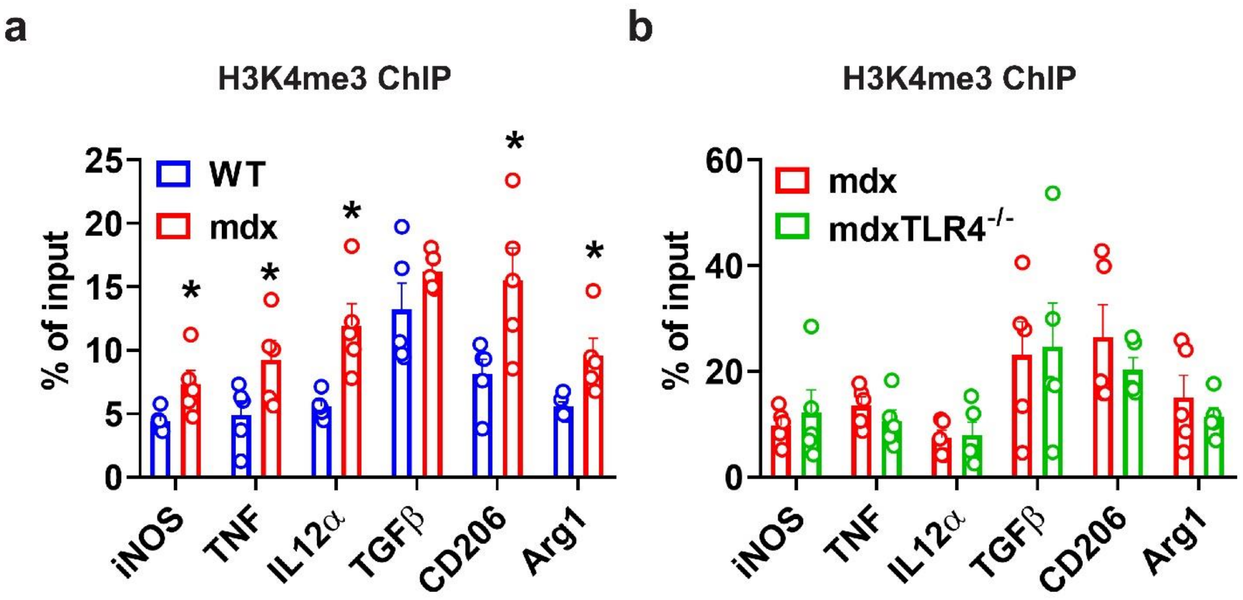

Supplementary Figure 7: H3K4me3 promoter occupancy in BMDM. ChIP-qPCR was performed to determine promoter region occupancy of H3K4me3 on both M1 and M2 marker genes in (**a**) WT versus mdx BMDM (n=5 per group) and (**b**) mdx versus mdxTLR4$^{-/-}$ BMDM (n=5 per group). IgG antibody was used as a control for non-specific binding of antibody. Data represent means ± SEM of independent biological samples from different mice. *P<0.05 (unpaired t-test, two tailed). See Source Data file for the exact P values.

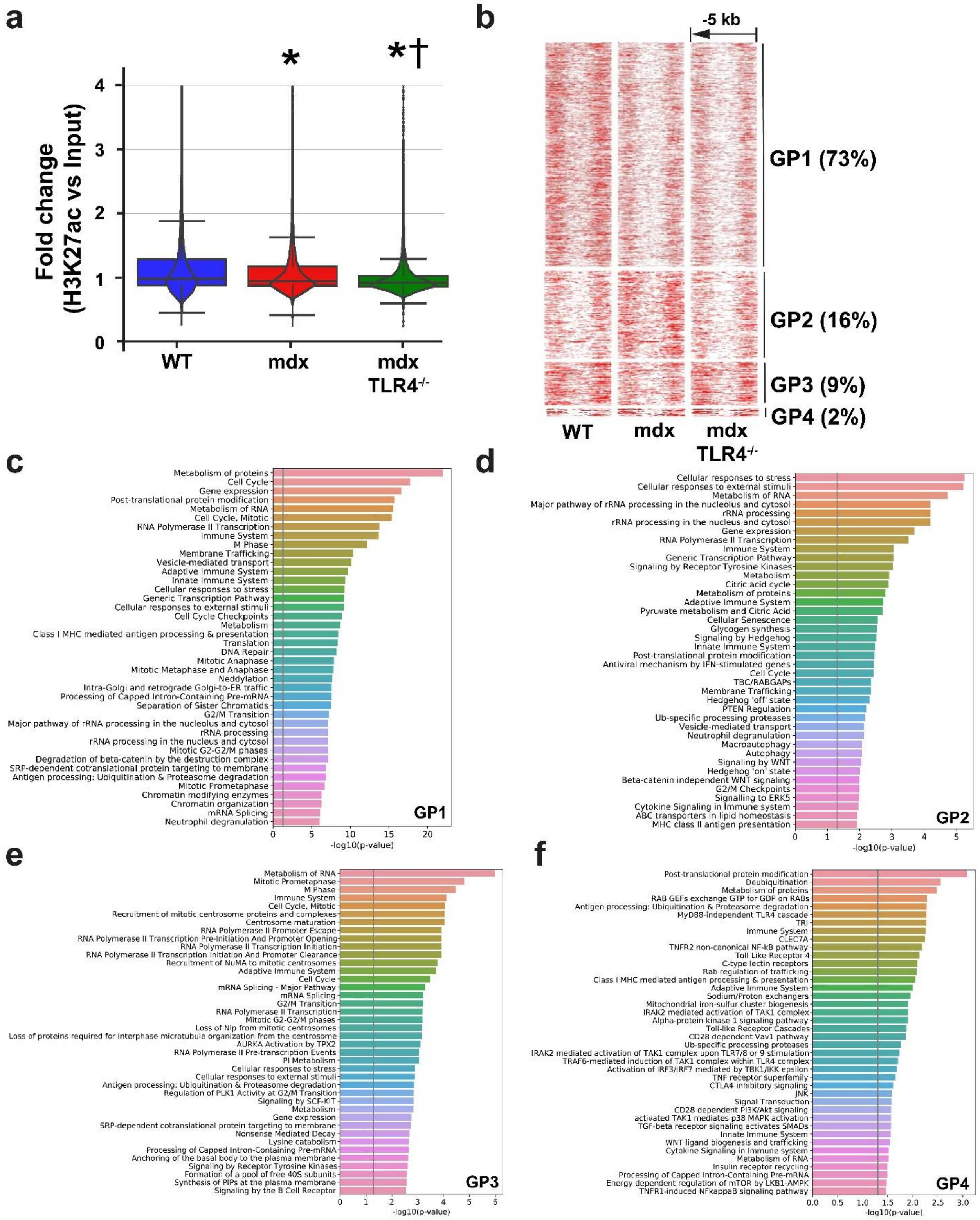

Supplementary Figure 8: Gene-based Pattern (GP) analysis of H3K27ac ChIP sequencing for WT, mdx and mdxTLR4$^{-/-}$ BMDM at the necrotic phase of disease.

(**a**) Violin/box plots representing normalized intensity fold change (H3K27ac vs. Input) in the region 5kb upstream to the transcription start site (TSS) of all genes across the whole genome; *P<0.0001 compared to WT BMDM, and ✝ P<0.0001 compared to the mdx group (two-sided Mann-Whitney U-test). Maxima and minima are shown at the extreme limits of the plot, box boundaries indicate the 25th and 75th percentiles, whiskers represent the 10th and 90th percentiles, and the horizontal line within the box denotes the median value. (**b**) Heatmaps showing different Gene-based Patterns (I=Increased and U=Unchanged relative to Input) in order of frequency: GP1 (IUU), GP2 (UIU), GP3 (IUI) and GP4 (UII) of the normalized H3K27ac read intensity (proportional to red color intensity) in the 5 kb promoter region of the genes. The values in parentheses indicate the percentages for each pattern. (**c**-**f**) Pathway enrichment analysis (Reactome database) for genes showing the GP1-4 configuration (see Supplementary Data 1 and 2 tables for the extended list of all Gene-based Patterns and enriched pathways, respectively). The vertical line indicates the cut-off P value = 0.05.

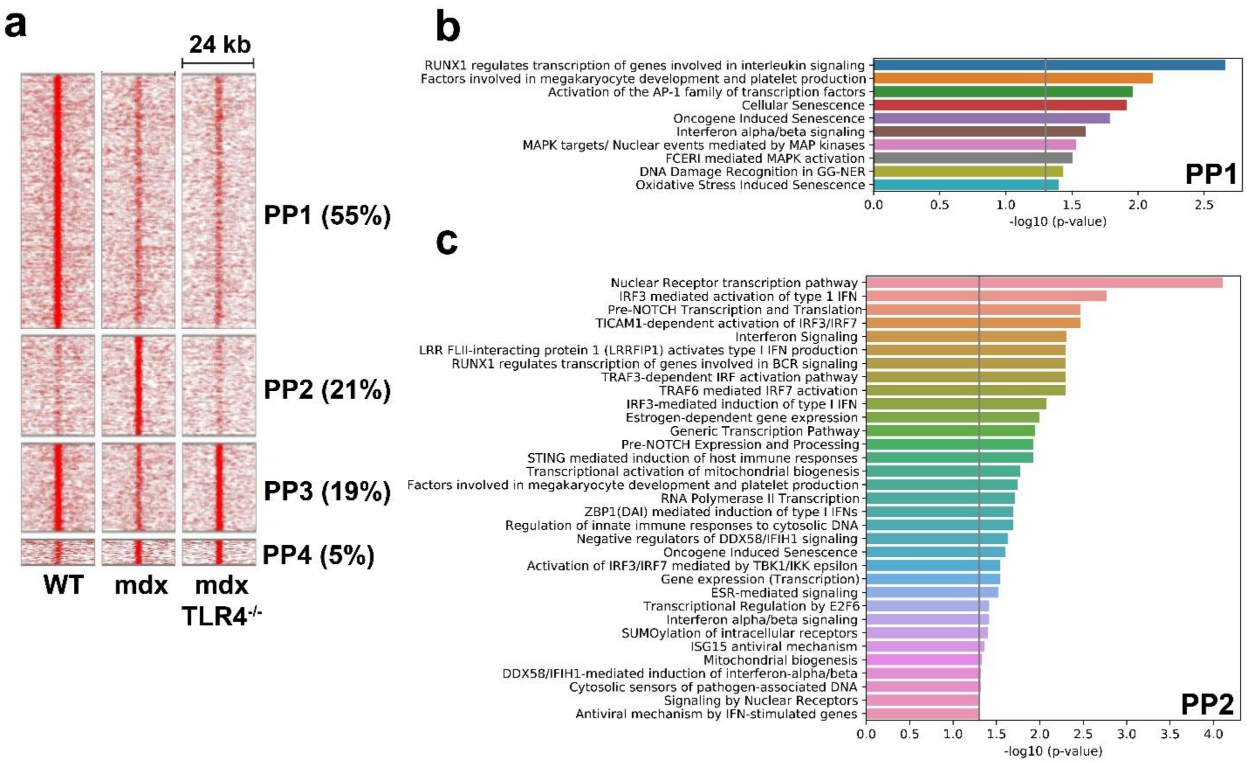

Supplementary Figure 9: Transcription factor enrichment analysis for H3K27ac in WT, mdx and mdxTLR4$^{-/-}$ BMDM. (**a**) Heatmaps showing different Peak-based Patterns (I=Increased and U=Unchanged relative to Input) in order of frequency: PP1 (IUU), PP2 (UIU), PP3 (IUI) and PP4 (UII) of the normalized H3K27ac read intensity (proportional to red color intensity) in the 24 kb nearby region around the peak. The values in parentheses indicate the percentages for each pattern. Transcription factor enrichment Reactome pathway analysis for (**b**) PP1 and (**c**) PP2 patterns are shown. Vertical lines indicate the cut-off P value = 0.05. The extended lists for these data can be found in Supplementary Data 4 and 5 tables.

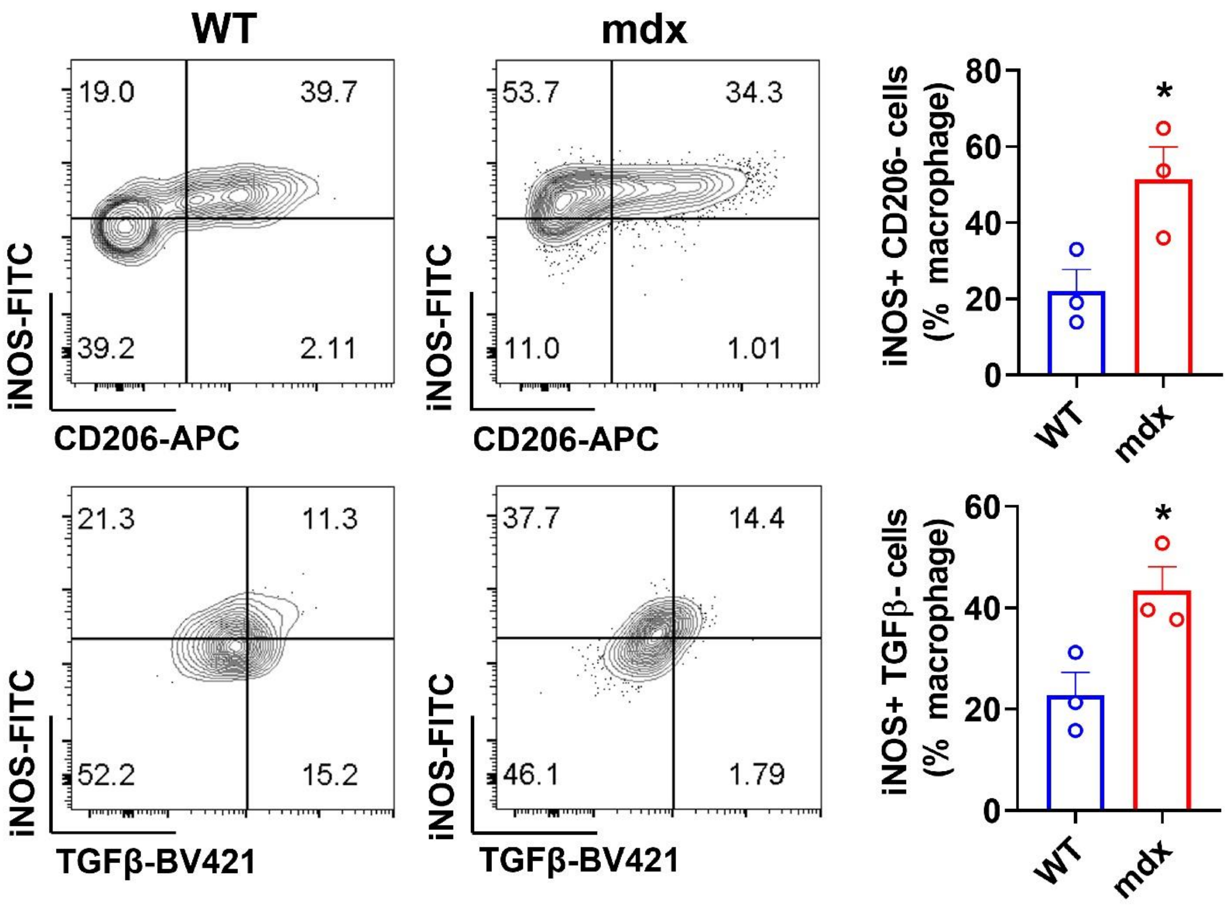

Supplementary Figure 10: Phenotype of macrophages in WT and mdx limb muscles. Tibialis anterior (TA) muscles from WT and mdx mice were processed and the percentages of pro-inflammatory macrophages (iNOS+ CD206- population, in upper panel and iNOS+ TGFβ- population, in lower panel) were determined by flow cytometry. Data are mean values ± SEM of independent biological samples from different mice (n=3/group). *P<0.05 (unpaired t-test, two-tailed). See Source Data file for the exact P values.

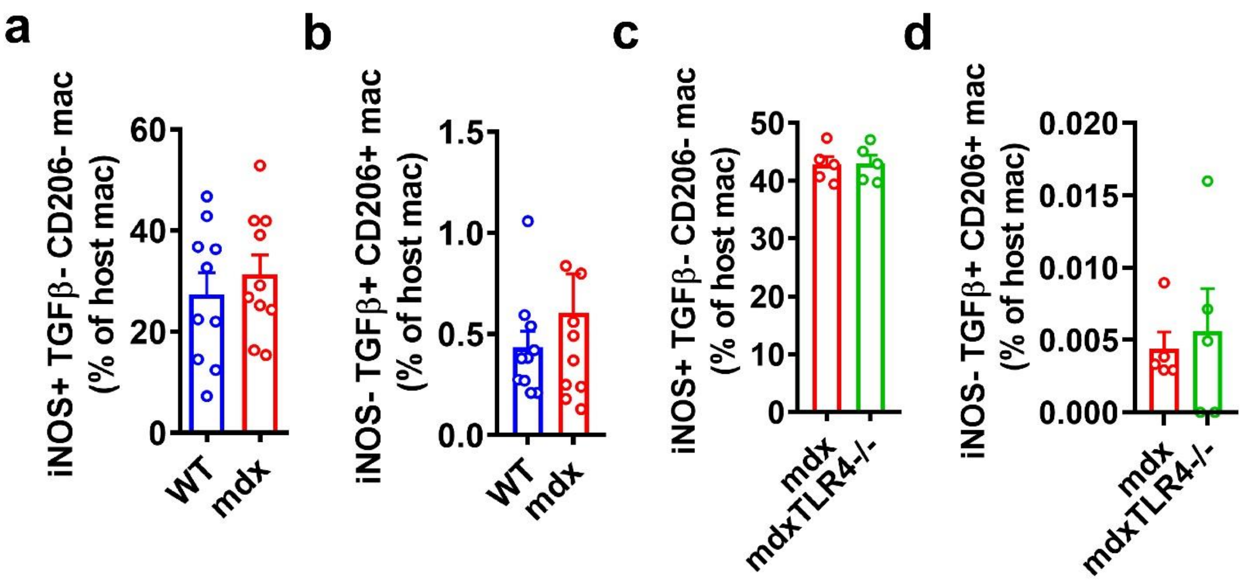

Supplementary Figure 11: Phenotype of host macrophages in injured muscles after adoptive transfer with WT or mdx BMDM. No significant differences in host (**a**) pro-inflammatory macrophages (iNOS+ CD206- TGFβ- population) (n=10/group) and (**b**) anti-inflammatory macrophages (iNOS- CD206+ TGFβ+ population) (n=9/group), were found in comparisons between mice adoptively transferred with either WT or mdx BM. Similarly, no significant differences in the host (**c**) pro-inflammatory macrophages (n=5/group) and (**d**) anti-inflammatory macrophages (n=5/group) were observed between groups adoptively transferred with either mdx or mdxTLR4$^{-/-}$ BM. All data are means ± SEM of independent biological samples from different mice. There are no significant differences between groups (unpaired t-test, two-tailed). See Source Data file for the exact P values.

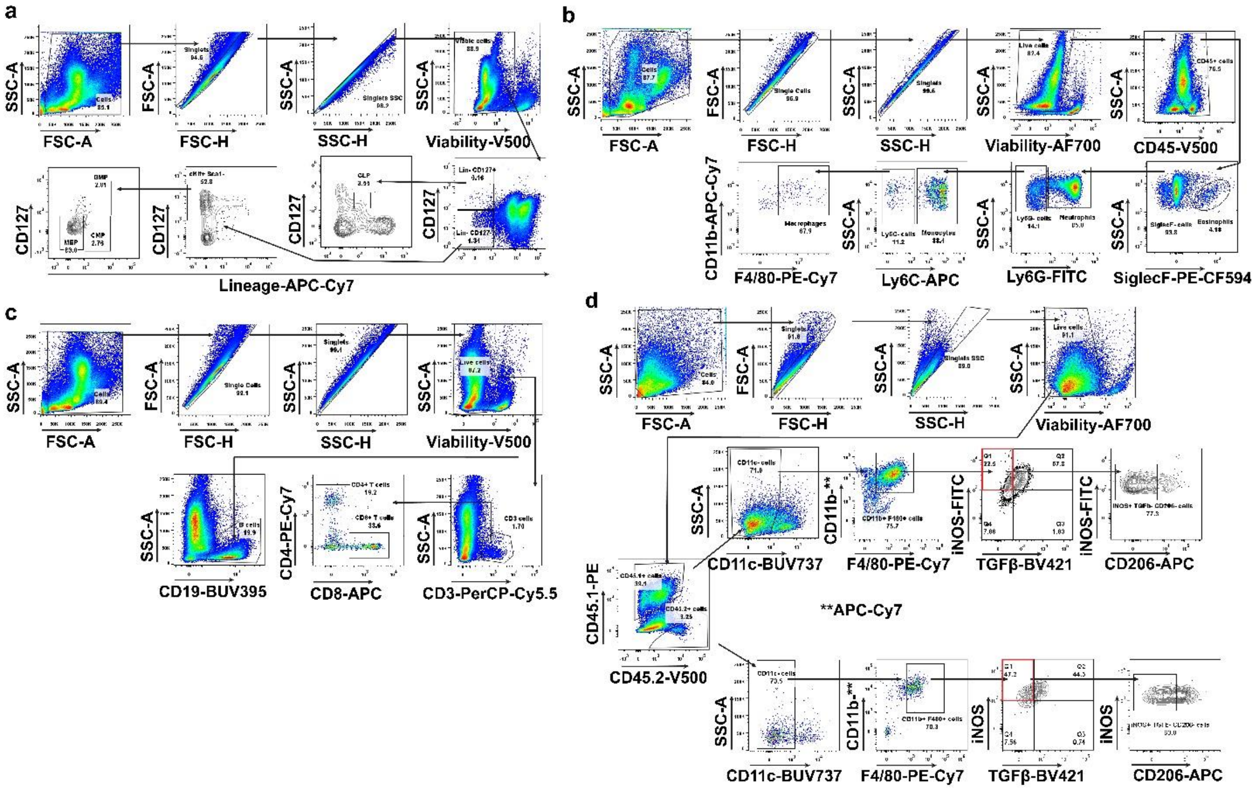

Supplementary Figure 12: Gating strategy. Flow cytometry gating strategies are shown for: (**a**) Hematopoietic progenitor cells, (**b**) Innate myeloid cells, and (**c**) Lymphocytes, in bone marrow. (**d**) Gating strategy for the analysis of pro- and anti-inflammatory macrophages in the injured host (CD45.1) limb muscles of mice after adoptive transfer with donor (CD45.2) bone marrow cells.
