# Supplementary material for: TLR4 is a regulator of trained immunity in a murine model of Duchenne muscular dystrophy
Source: Nat Commun. 2022 Feb 15;13:879. doi: 10.1038/s41467-022-28531-1 (PMC8847425; doi:10.1038/s41467-022-28531-1)
Supplement: Supplementary file 3 — Description of Additional Supplementary Files [file 41467_2022_28531_MOESM3_ESM.pdf]

Description of Additional Supplementary Files

Supplementary Data 1. List of genes associated with significantly different histone modification patterns (GP1-GP4) in bone marrow-derived macrophages (BMDM) from wild-type (WT), mdx, and mdxTLR4-/- mice. The left and right panels show the histone modification patterns for H3K27me3 and H3K27ac, respectively.

Supplementary Data 2. Biological pathway enrichment analysis (Reactome informatics database) and associated gene lists for the different histone modification patterns (GP1-GP4) found in bone marrow-derived macrophages (BMDM) from wild-type (WT), mdx, and mdxTLR4-/- mice. The left and right panels show the pathway analysis for H3K27me3 and H3K27ac, respectively.

Supplementary Data 3. List of genes selected a priori to reflect potential pro-inflammatory, anti-inflammatory, and pro-fibrotic functions of bone marrow-derived macrophages (BMDM) from wild-type (WT), mdx, and mdxTLR4-/- mice.

Supplementary Data 4. List of peak regions within the genome exhibiting significantly different histone modification patterns (PP1-PP4) in bone marrow-derived macrophages (BMDM) from wild-type (WT), mdx, and mdxTLR4-/- mice. The left and right panels show the peak-based histone modification patterns for H3K27me3 and H3K27ac, respectively.

Supplementary Data 5. Transcription factor (TF) pathway enrichment analysis for the dominant peak-based histone modification patterns (PP1-PP2) found in bone marrow-derived macrophages (BMDM) from wild-type (WT), mdx, and mdxTLR4-/- mice. The left and right panels show the TF pathway analysis for H3K27me3 and H3K27ac, respectively.

Supplementary Data 6. PCR primer sequences used to evaluate mRNA expression or histone modifications of gene promoters for prototypical pro-inflammatory and anti-inflammatory genes.
