# Supplementary material for: TLR4 is a regulator of trained immunity in a murine model of Duchenne muscular dystrophy
Source: Nat Commun. 2022 Feb 15;13:879. doi: 10.1038/s41467-022-28531-1 (PMC8847425; doi:10.1038/s41467-022-28531-1)
Supplement: Supplementary file 10 — Reporting summary [file 41467_2022_28531_MOESM10_ESM.pdf]

nature portfolio

Corresponding author(s): Prof. Basil J Petrof
Last updated by author(s): Dec 16, 2021

Reporting Summary

Nature Portfolio wishes to improve the reproducibility of the work that we publish. This form provides structure for consistency and transparency in reporting. For further information on Nature Portfolio policies, see our Editorial Policies and the Editorial Policy Checklist.

Statistics

For all statistical analyses, confirm that the following items are present in the figure legend, table legend, main text, or Methods section.

n/a	Confirmed	
☐	☒	The exact sample size (*n*) for each experimental group/condition, given as a discrete number and unit of measurement
☐	☒	A statement on whether measurements were taken from distinct samples or whether the same sample was measured repeatedly
☐	☒	The statistical test(s) used AND whether they are one- or two-sided<br>*Only common tests should be described solely by name; describe more complex techniques in the Methods section.*
☒	☐	A description of all covariates tested
☐	☒	A description of any assumptions or corrections, such as tests of normality and adjustment for multiple comparisons
☐	☒	A full description of the statistical parameters including central tendency (e.g. means) or other basic estimates (e.g. regression coefficient) AND variation (e.g. standard deviation) or associated estimates of uncertainty (e.g. confidence intervals)
☐	☒	For null hypothesis testing, the test statistic (e.g. *F*, *t*, *r*) with confidence intervals, effect sizes, degrees of freedom and *P* value noted<br>*Give P values as exact values whenever suitable.*
☒	☐	For Bayesian analysis, information on the choice of priors and Markov chain Monte Carlo settings
☒	☐	For hierarchical and complex designs, identification of the appropriate level for tests and full reporting of outcomes
☒	☐	Estimates of effect sizes (e.g. Cohen's *d*, Pearson's *r*), indicating how they were calculated

Our web collection on statistics for biologists contains articles on many of the points above.

Software and code

Policy information about availability of computer code

Data collection	Flowcytometry: BD FacsDiva version 8 software<br>ChIP seq: Hi-seq4000<br>qPCR: Applied Biosystems StepOnePlus™ Real-Time PCR Systems (v. 2.3)<br>Seahorse assay: Seahorse Wave software (v. 2.6.1.53)
Data analysis	Flowcytometry: FlowJo version 10<br>Data presentation and statistical analysis: Graphpad version 6.01<br>Western blot quantification: Image Lab version (v.6.0.1) build 34<br>ChiP Seq data analysis: trimming reads: fastp (v. 0.20.1), mapping reads: bowtie2 (v. 2.3.4.1) and peak calling: SICER2 (v. 1.0.2)<br>quantify the read intensity across the genome: deeptools (v. 3.5.0)<br>Seahorse assay: Seahorse Wave software (v. 2.6.1.53) and microsoft excel

For manuscripts utilizing custom algorithms or software that are central to the research but not yet described in published literature, software must be made available to editors and reviewers. We strongly encourage code deposition in a community repository (e.g. GitHub). See the Nature Portfolio guidelines for submitting code & software for further information.

Data

Policy information about availability of data

All manuscripts must include a data availability statement. This statement should provide the following information, where applicable:

- Accession codes, unique identifiers, or web links for publicly available datasets
- A description of any restrictions on data availability
- For clinical datasets or third party data, please ensure that the statement adheres to our policy

The data that support the findings of this study are available from the corresponding author upon reasonable request. ChIP-seq generated in this study have been deposited in the Gene Expression Omnibus database under accession number GSE190307. We provide a data server to enable interactive queries of the H3K27me3 status for any gene list (https://junding.lab.mcgill.ca/research/basil/EnrichmentServer/). Similarly, a webserver for interactive exploration of H3K27ac is also available on (https://junding.lab.mcgill.ca/research/basil/H3K27ac/EnrichmentServer/).Source data are provided with this paper.

Field-specific reporting

Please select the one below that is the best fit for your research. If you are not sure, read the appropriate sections before making your selection.

[x] Life sciences [ ] Behavioural & social sciences [ ] Ecological, evolutionary & environmental sciences

For a reference copy of the document with all sections, see nature.com/documents/nr-reporting-summary-flat.pdf

Life sciences study design

All studies must disclose on these points even when the disclosure is negative.

Sample size	No sample-size calculation was performed. Experiments were performed with sufficient power to achieve statistical significance. All experiments were performed with the number of mice indicated in the corresponding figure legend.
Data exclusions	Data exclusions was considered to be the one with the outlier values. Possible outlier was calculated on each graph by Graphpad. Else, no other data were excluded from the analysis.
Replication	The results were concluded with experiments that were repeated for at least 2 times.
Randomization	All groups were aged and sexed matched. In our study, sex for all mice are male. No randomization was performed for in vivo studies. For in vitro studies, randomization was not required since a homogeneous pool of BMDM were used with the indicated conditions.
Blinding	All researchers performed, acquired and analyzed the experiments and were not blinded because the experiments we implemented in this study was not applicable for blinding.

Reporting for specific materials, systems and methods

We require information from authors about some types of materials, experimental systems and methods used in many studies. Here, indicate whether each material, system or method listed is relevant to your study. If you are not sure if a list item applies to your research, read the appropriate section before selecting a response.

Materials & experimental systems

n/a	Involved in the study	
[ ]	[x]	Antibodies
[ ]	[x]	Eukaryotic cell lines
[x]	[ ]	Palaeontology and archaeology
[ ]	[x]	Animals and other organisms
[x]	[ ]	Human research participants
[x]	[ ]	Clinical data
[x]	[ ]	Dual use research of concern

Methods

n/a	Involved in the study	
[ ]	[x]	ChIP-seq
[ ]	[x]	Flow cytometry
[x]	[ ]	MRI-based neuroimaging

Antibodies

Antibodies used	Antibodies for flowcytometry were purchased from BD otherwise stated and used at 1:100 dilution:<br>PE-labelled anti-CD45.1 (clone A20), #553776<br>V500 labelled anti-CD45.2 (clone 104), #562130<br>BUV 395 labelled anti-CD45.2 (clone 104), #564616<br>BUV737 labelled anti-CD11c (clone HL3, 1:50), #612797<br>APC-Cy7 labelled anti-CD11b (clone M1/70), BioLegend #101226

PE-Cy7 labelled anti-F4/80 (clone BM8), BioLegend #123114
biotin conjugated CD5 (clone 53-7.3), BioLegend #100604, anti-Ly6G/C (clone RB6-8C5), BioLegend #553124, anti-CD11b (clone M1/70), #101204, anti-CD4 (clone RM4-5) #553309, anti-CD8a (clone 53-6.7) #553028, anti-CD45R (clone RA3-6B2) #553086,
Streptavidin conjugated APC-Cy7 #554063,
BV785 labelled anti-CD127 (clone A7R34) #135037 ,
PE-Cy7 labelled anti-Sca-1 (clone D7) Biolegend #108114,
APC labelled anti-c-Kit (clone 2B8), #561074
FITC labelled anti-CD34 (clone RAM34) #553733,
PerCp-Cy5.5 labelled anti-CD16/32 (clone 93) #560540,
PerCP-Cy5.5 labelled anti-CD3 (clone 145-2C11) #551163,
PE-Cy7 labelled anti-CD4 (clone 53-6.7) Biolegend #100722,
APC labelled CD8 (clone 53-6.7) #553035,
BUV395 labelled anti-CD19 (clone 1D3) #563557,
FITC labelled anti-Ly6G (Clone 1A8,) BioLegend #127606,
APC labelled anti-Ly6C (Clone HK1.4,) BioLegend #128016 ,
PE-CF594 labelled anti Siglec-F (Clone E50-2440) #562757,
FITC labelled anti-iNOS (clone 6/ iNOS/NOS Type II) #610330,
BV421 labelled anti-TGFβ (clone TW7-16B4, 1:200) #565638
Antibodies for Westernblot, purchased from Cell signaling otherwise stated and used at 1:1000 dilution: p-STAT1 (clone 58D6) (#9167), total STAT1 (#9172) , p-STAT3 (#9131), total STAT3 (#9139), p-STAT6 (#9361), total STAT6 (#9362), Arginase-1 (#93668), iNOS Monoclonal (6/iNOS/NOS Type II, 1:500 BD bioscience #610329), HRP-conjugated secondary anti-mouse antibody (Promega, #W4021, 1:5000 dilution) and HRP-conjugated secondary anti–rabbit antibody (Promega, #W4011, 1:2500 dilution)
Antibodies for ChIP assay/seq were used at the concentration recommended by the manufacturer: H3K27me3 (#9733, Cell Signaling), H3K4me3 (ab8580, Abcam), H3K27Ac (39133, Active Motif), IgG (#3900, Cell Signaling)

Validation

All antibodies for flowcytometry were validated by manufacturer. Additional validation were performed using fluorescence Minus One (FMO) controls for each samples.
All antibodies for western blot were validated in laboratory using known positive control and molecular weight of protein in gel.
As per manufacturer's product information, rabbit anti-p-STAT1 antibody (#9167, Cell Signaling Technology) has validated to detect endogenous protein of mouse p-STAT1 by western blot analysis
As per manufacturer's product information, rabbit anti-STAT1 antibody (#9172, Cell Signaling Technology) has validated to detect endogenous protein of mouse total STAT1 by western blot analysis
As per manufacturer's product information, rabbit anti-p-STAT3 antibody (#9131, Cell Signaling Technology) has validated to detect endogenous protein of mouse p-STAT3 by western blot analysis
As per manufacturer's product information, mouse anti-STAT3 antibody (#9139, Cell Signaling Technology) has validated to detect endogenous protein of mouse STAT3 by western blot on extracts from NIH/3T3 cells
As per manufacturer's product information, rabbit anti-p-STAT6 antibody (#9361, Cell Signaling Technology) has validated to detect endogenous protein of mouse p-STAT6 by western blot analysis
As per manufacturer's product information, rabbit anti-STAT6 antibody (#9362, Cell Signaling Technology) has validated to detect endogenous protein of mouse STAT6 by western blot on extracts from 3T3/LI cells
As per manufacturer's product information, rabbit anti-Arginase-1 antibody (#93668), Cell Signaling Technology) has validated to detect endogenous protein of mouse arginase-1 by western blot on extracts from mouse liver
As per manufacturer's product information, mouse anti-iNOS antibody (#93668, BD BBCell Signaling Technology) has validated to detect endogenous protein of mouse iNOS by western blot on extracts from mouse macrophages
As per manufacturer's product information, mouse anti-actin antibody (#A2228, Sigma) has validated to detect endogenous protein of mouse actin by western blot
The goat anti-Rabbit IgG (H+L), HRP Conjugate (W4011, Promega) has been validated to be used as a secondary antibody for western blot
The goat anti-mouse IgG (H+L), HRP Conjugate (W4021, Promega) has been validated to be used as a secondary antibody for western blot
As per manufacturer's product information, Rabbit mAb against mouse H3K27me3 (#9733, Cell Signalling Technology) for ChIP sequencncing has been validated using SimpleChIP® Enzymatic Chromatin IP Kits
As per manufacturer's product information, Rabbit polyclonal antibody against mouse H3K4me3 (#ab8580, Abcam) has been extensively validated for ChIP assay
As per manufacturer's product information, Rabbit polyclonal antibody against mouse H3K27Ac (#39133, Active motif) was validated for ChIP-Seq

Eukaryotic cell lines

Policy information about cell lines

Cell line source(s)

BMDM primary cell line was generated by culturing whole bone marrow cells from mice in L929 conditioned media. L929 cells were purchased from American Type Culture Collection

Authentication

Growth rate and morphology of cells were checked frequently, but these primary cell lines were not authenticated.

Mycoplasma contamination

BMDM primary culture were not tested for mycoplasma

Commonly misidentified lines
(See ICLAC register)

None of the commonly misidentified lines were used

Animals and other organisms

Policy information about studies involving animals; ARRIVE guidelines recommended for reporting animal research

Laboratory animals	Animals (C57BL/6 mice, mdx4cV and TLR4-/-), were purchased from Jackson Laboratories and inhouse bred for experiments. mdxTLR4-/- mice were generated as per previous studies (Giordano et al 2015) in the animal facility. Studies were performed in male aged-matched mice between 4weeks to 6months of age.
Wild animals	The study did not involve wild animals.
Field-collected samples	Study did not involved samples collected from the field
Ethics oversight	All animal studies were conducted in accordance with the guidelines of and approved by the Animal Research Ethics Board of McGill University.

Note that full information on the approval of the study protocol must also be provided in the manuscript.

ChIP-seq

Data deposition

☐ Confirm that both raw and final processed data have been deposited in a public database such as GEO.

☒ Confirm that you have deposited or provided access to graph files (e.g. BED files) for the called peaks.

Data access links<br>*May remain private before publication.*	For H3K27me3: https://junding.lab.mcgill.ca/research/basil/EnrichmentServer/<br>For H3K27ac: https://junding.lab.mcgill.ca/research/basil/H3K27ac/EnrichmentServer/
Files in database submission	ChIP-seq data will be uploaded in the public database after the acceptance of the manuscript.
Genome browser session<br>(e.g. UCSC)	The above mentioned link includes pathway for the Genome browser session for both epigenetic mark.

Methodology

Replicates

For each of the conditions (WT, mdx, mdxTLR4-/-), we have two replicates for both H3K27mc3 and H3K27ac ChIP-seq measurements.

Sequencing depth

All the replicates ChIP-seq samples are merged for the downstream analysis. All the reads in this study are single-ended. Please find following for the detailed sequencing depth information for each sample

1) H3K27me3

WT replicate r1: total # of reads= 21434711, mapped %=95.34%, read length=50
WT replicate r2: total # of reads=23132293, mapped %=95.87%, read length=50
WT input r1: total # of reads=24421362, mapped %=95.42%, read length=50
WT input r2: total # of reads=6863399, mapped %=93.34%, read length=50

mdx replicate r1: total # of reads=18442400 mapped %=94.26%, read length=50
mdx replicate r2: total # of reads=20714786 mapped %=94.08%, read length=50
mdx input r1: total # of reads=18119644, mapped %=93.72%, read length=50
mdx input r2: total # of reads=27067445, mapped %=93.01%, read length=50

mdxTLR4-/- replicate r1: total # of reads=30599286 mapped %=93.31%, read length=50
mdxTLR4-/- replicate r2: total # of reads=22437711 mapped %=95.01%, read length=50
mdxTLR4-/- input r1: total # of reads=23864757, mapped %=92.12%, read length=50
mdxTLR4-/- input r2: total # of reads=15014705, mapped %=90.77%, read length=50

2) H3K27ac

WT replicate r1: total # of reads= 5276177, mapped %=86.2%, read length=75
WT replicate r2: total # of reads=1493419, mapped %=77.56%, read length=75

mdx replicate r1: total # of reads=4286907, mapped %=91.23%, read length=75
mdx replicate r2: total # of reads=810590, mapped %=82.41%, read length=75

mdxTLR4/- replicate r1: total # of reads= 3604992, mapped %=64.22%, read length=75

Input: total # of reads=7578732, mapped %=97.86%, read length=75

Antibodies	Please see this information above on antibodies section
Peak calling parameters	All raw reads were trimmed using fastp (v 0.20.1) with the default parameter. The trimmed reads were mapped to the mouse

	reference genome mm10 using bowtie2. Broad peaks were called using SICER2 with the default parameters.
Data quality	Under FDR=0.01, we identified 28143 peaks for WT, 41780 peaks for mdx, and 36487 peaks for mdxTLR4-/- respectively using SICER2 with the default parameters.
Software	trimming reads: fastp (v. 0.20.1)<br>mapping reads: bowtie2 (v. 2.3.4.1)<br>peak calling: SICER2 (v. 1.0.2)

Flow Cytometry

Plots

Confirm that:

- [x] The axis labels state the marker and fluorochrome used (e.g. CD4-FITC).
- [x] The axis scales are clearly visible. Include numbers along axes only for bottom left plot of group (a 'group' is an analysis of identical markers).
- [x] All plots are contour plots with outliers or pseudocolor plots.
- [x] A numerical value for number of cells or percentage (with statistics) is provided.

Methodology

Sample preparation	For Bone marrow: Bone marrow from mice was isolated and resuspended 10^6 cells into PBS with 0.5% BSA.<br>For muscle, single cell suspensions were obtained by incubating minced muscles in 0.2% collagenase (Roche) at 37oC for 45 minutes followed by filtering with a 70-µm cell strainer. For flow cytometry, 10^6 cells were resuspended in PBS containing 0.5 % BSA.
Instrument	BD LSRFortessa X-20 was used for data collection.
Software	BD FACSDiva™ Software was used to collect the data and FlowJo™ V10 Software was used to make an analysis
Cell population abundance	Our studies do not contain facs-sorting experiments.
Gating strategy	FSC/SSC were gated based on the cell size compared to a standard beads. Positive population were determined in reference with the controls of the unstained sample for that particular population. Details about gating has been described in methods sections and the strategy is provided in the supplementary figure.

- [x] Tick this box to confirm that a figure exemplifying the gating strategy is provided in the Supplementary Information.
